# 132. Carbapenem-resistant Enterobacteriaceae colonization in a Neonatal Intensive Care Unit in Bogotá, Colombia: Characterization and clinical outcomes

**DOI:** 10.1093/ofid/ofac492.210

**Published:** 2022-12-15

**Authors:** Juan Pablo Londono-Ruiz, Martha Isabel Alvarez-olmos, Diana Carolina Medina-ramos, Gloria Amparo Troncoso-Moreno, Jose Antonio de la Hoz-Valle

**Affiliations:** Universidad El Bosque, Bogota, Distrito Capital de Bogota, Colombia; Fundación Cardioinfantil, Bogota, Distrito Capital de Bogota, Colombia; Fundación Cardioinfantil, Bogota, Distrito Capital de Bogota, Colombia; Fundación Cardioinfantil, Bogota, Distrito Capital de Bogota, Colombia; Universidad El Bosque- Fundacion Santa Fe de Bogota, Bogota, Distrito Capital de Bogota, Colombia

## Abstract

**Background:**

Carbapenem-resistant Enterobacteriaceae (CRE) infections is a global public health problem, especially among vulnerable populations. Studies exploring these outcomes in neonates colonized with CRE are scarce. The aim is to explore differences in clinical outcomes between CRE-colonized versus CRE-non-colonized neonates.

**Methods:**

A retrospective, observational, and longitudinal cohort study was performed for surveillance of CRE colonization in the neonates admitted between 2018-2021 to NICU in Bogotá, Colombia.

**Results:**

From 462 clinical records, 21 (4,5%) were CRE colonized neonates. We found a difference in median gestational age to birth (34 weeks in CRE-colonized vs 37 weeks in non-CRE colonized *p 0,018*), any previous antibiotic therapy (71% vs 25% *p 0,005*), carbapenem use before admission (19% vs 1,6% *p 0,01*), CRE isolation in any sterile fluid during sepsis episodes (14% vs 0% *p 0,004*), use of empiric carbapenem therapy in sepsis episodes (42% vs 6,3% *p 0,0003*), empiric therapy for CRE infection (28% vs 6,3% *p 0,01*), and hospital length of stay (22 vs 11 days *p 0,007*). We did not find difference in sepsis episodes (66% vs 39% *p 0,058*) and mortality (4,8% vs 7,9%) among both groups.

**Conclusion:**

Gestational age, prior use of any antibiotic or carbapenems, CRE isolation in sterile fluid during sepsis episodes, and higher length of stay were more frequently identified among CRE-colonized neonates versus CRE non-colonized neonates.

**Disclosures:**

**All Authors**: No reported disclosures.

